# The reliability of the general functioning scale in Norwegian 13–15-year-old adolescents and association with family dinner frequency

**DOI:** 10.1186/s12937-019-0447-1

**Published:** 2019-03-27

**Authors:** Solveig E. S. Hausken, Hanne C. Lie, Nanna Lien, Ester F. C. Sleddens, Elisabeth L. Melbye, Mona Bjelland

**Affiliations:** 1 Department of Nutrition, Institute of Basic Medical Sciences, Faculty of Medicine, University of Oslo, P.O.Box 1046, Blindern, NO-0316 Oslo, Norway; 2 Department of Behavioural Sciences in Medicine, Institute of Basic Medical Sciences, Faculty of Medicine, University of Oslo, Oslo, Norway; 3 Department of Health Promotion, School of Nutrition and Translational Research in Metabolism (NUTRIM), Maastricht University Medical Center, Maastricht, The Netherlands; 4 Faculty of Social Sciences, Norwegian School of Hotel Management, University of Stavanger, Stavanger, Norway

**Keywords:** Family functioning, Family dinners, Adolescents

## Abstract

**Background:**

Family environment is crucial to the development of health behaviors into adolescence and adulthood. The aims of this study were (1) to explore the reliability of the General Functioning Scale (GFS) among Norwegian 13-15-year-olds, and (2) to assess whether family functioning reported by adolescents was associated with family dinner frequency.

**Methods:**

In total 440 secondary-school students were invited to participate in this cross-sectional web-based questionnaire survey, with 54 participating in the test-retest study. Test-retest and internal consistency were assessed for the 12-item GFS-scale. Associations between family functioning and family dinner frequency were tested using multiple logistic regression.

**Results:**

The GFS had high internal consistency (corrected item-total correlations ranging from 0.40 to 0.65, Cronbach’s α = 0.85), and excellent test–retest reliability (intra-class correlation coefficient = 0.83). In the logistic regression model, a higher score on GFS (poorer family functioning) was associated with a reduced likelihood of having dinner together on a daily basis (i.e., 6–7 times per week, OR = 0.36, CI = 0.20–0-64) after adjusting for age, gender, ethnicity, living situation and parental education level.

**Conclusions:**

The GFS had high reliability. As poorer family functioning was associated with less frequent family dinners, the family environment may be an important (contextual) target to influence adolescent health behaviors. It would be of interest to further explore the role of family functioning in relation to adolescents’ dietary habits, besides shared family meals, and to reveal the mechanisms underlying such relationships.

**Electronic supplementary material:**

The online version of this article (10.1186/s12937-019-0447-1) contains supplementary material, which is available to authorized users.

## Background

Studies have shown that health behaviors in adolescence can be maintained into adulthood [1, 2]. Adolescence is therefore considered an important developmental period for establishing favorable health behaviors such as healthy eating habits. The family and the home environment are important settings for influencing and shaping children’s and youths’ eating habits [3]. Factors such as parenting style and parenting practices (e.g., modeling behavior) have been well studied in relation to adolescents’ food consumption and/or weight status [4, 5]. However, these factors do not account for the overall effect of the family environment on adolescents’ eating habits. A sociocultural factor that has been studied to a limited degree is family functioning [6]. Family functioning refers to the relationship within the family, the social connectedness and closeness of the family, as well as the level of problem solving and behavioral control [7]. Previous research reports that family functioning can be an important protective factor against adolescents’ fast food intake, lack of physical activity, disordered eating, sedentary behavior and low breakfast frequency [5, 6]. Furthermore, an American study [6] highlights that it is important to identify whether, and how, family functioning is associated with other behavioral outcomes, like family meals. According to a systematic review by Harrison et al. [8], frequent family meals are inversely associated with negative behaviors (e.g., disordered eating, alcohol, substance use) and positively related to increases of self-esteem and school success. Exactly how family meals are related to family functioning is, however, yet to be determined.

Family functioning becomes visible during family meal activities, such as in the planning, preparation, and eating situation [5]. The family meal may promote family conversation about food, give parents an opportunity to model healthy eating and build a sense of community and belonging [9, 10]. Even if adolescence is a time for increased independence and spending more time away from home, research has shown that family meals are perceived as a positive experience by both parents and adolescents [9]. Research also underscores the importance of eating family meals (mainly dinner) on a regular basis as this is associated with lowered odds of poor diet quality and breakfast skipping [10].

Few studies assess both family functioning and the frequency of family meals. One study from America found that a good family functioning was associated with more frequent family meals, even after adjusting for age, socioeconomic status and race/ethnicity [6]. Furthermore, to our knowledge, no Norwegian study has explored family functioning using a Norwegian version of the General Functioning Scale (GFS) in relation to family meal frequency. Dinner is the most important family meal among Norwegian adolescents according to a national survey from 2000 [11], and therefore dinner was chosen as the measure of family meals in this study.

The aim of the present study was two-fold: 1) to explore the reliability of the General Functioning Scale (GFS) in Norwegian 13–15-year-old adolescents, and [2] to assess whether family functioning reported by adolescents is associated with family dinner frequency.

## Methods

### Sample and data collection

The participants in this study were students from a convenience sample of five secondary schools in three Norwegian counties (Akershus, Oslo and Østfold). In total, 1136 adolescents (13–15-year old) were invited to take part in the cross-sectional study, of which 440 (39%) participated. Of these, 204 were invited to engage in a test-retest study, of which 54 adolescents (26%) participated. For practical reasons the retest was conducted among pupils in only one of the schools participating.The test and retest were conducted 10–14 days apart. Informed parental consent was obtained from all participants. The adolescents filled in a web-based questionnaire at school. Details about the questionnaire development are presented elsewhere [12]. All measures were assessed by self-report, except parental education, which was reported by the parents in the parental consent form. A group of experts (five professors, four postdoctoral researchers and one lecturer with different backgrounds related to family processes and dietary habits) assessed the content and face validity of the applied measures. The Norwegian Social Science Data Services has approved the study and The Regional Committees for Medical and Health Research Ethics has been informed, but no approval was needed.

### Family dinner frequency

Frequency of family dinners was assessed by one question: “How often does your mother and/or father usually sit down and eat dinner with you?” with eight categories ranging from never/seldom to seven times a week [9]. The family dinner variable was not normally distributed; most of the adolescents ate dinner together with their parent(s) 6 or 7 times per week (80.5%). Therefore, responses were dichotomized into “0-5 times a week” and “6-7 times a week”.

### Family functioning

Family functioning was measured with a Norwegian version of the GFS, a 12-item scale extracted from the McMaster Family Assessment Device (FAD) assessing the overall family functioning (see Table 1 for items) [13, 14]. Details about the translation process of the GFS are presented elsewhere [12]. The response categories ranged from 1 (*Strongly agree*) to 4 (*Strongly disagree*), where the sum of scores was divided by 12 to give a total average score ranging from 1.0 to 4.0. A higher score indicates poorer family functioning. Previous research has shown good reliability and construct validity for the GFS in racially/ethnically and socioeconomically diverse populations [13, 15]. Recent research on adolescents have showed excellent internal consistency of the GFS among Armenian adolescents (α = .80) [16], and high test-retest reliability among Chinese adolescents (r = .77) [17]. Furthermore, the scale showed a high internal consistency in different Chinese adolescent samples and acceptable convergent and construct validity [17]. In addition to support for the scale’s reliability and validity among adolescents, the two mentioned studies also supports the cultural appropriateness of the scale [16, 17].

**Table 1:** Scale measurement properties of the General Functioning Scale (GFS)

	Full sample *n* = 399				Test-retest *n* = 45
Item	Mean	SD	CITC^b^	α^c^	ICC^d^
**Total score General Functioning Scale** ^a^	1.72	(0.56)	–	0.85	0.83
Planning family activities is difficult because we misunderstand each other (reversed).	1.79	(0.79)	0.52		
In times of crisis, we turn to each other for support.	1.79	(0.73)	0.44		
We cannot talk to each other about the sadness we feel (reversed).	1.87	(0.91)	0.40		
Individuals are accepted for what they are.	1.68	(0.77)	0.42		
We avoid discussing our fears and concerns (reversed).	2.03	(0.76)	0.52		
We express feelings to each other.	1.84	(0.79)	0.53		
There are lots of bad feelings in our family (reversed).	1.57	(0.74)	0.52		
We feel accepted for what we are.	1.43	(0.62)	0.65		
Making decisions is a problem for our family (reversed).	1.76	(0.74)	0.58		
We are able to make decisions about how to solve problems.	1.66	(0.65)	0.62		
We do not get along well together (reversed).	1.54	(0.78)	0.52		
We confide in each other.	1.66	(0.66)	0.61		

^a^Answer categories ranging from 1 (strongly agree) to 4 (strongly disagree). The total score is then divided by the number of items on the subscale giving a total averaged score ranging from 1.0 (healthy functioning) to 4.0 (unhealthy functioning)

^b^Corrected Item-Total Correlation for assessment of internal consistency

^c^Cronbach’s alpha for assessment of internal consistency

^d^Intra-class correlation assessing test-retest reliability

### Covariates

Gender was categorized into “boy” and “girl”. Parental education level was categorized into three levels: “12 years or less” (level 1), “between 13-16 years” (level 2) and “more than 16 years” (level 3). Participant ethnicity was categorized as “Norwegian” or “other”, where other was defined as those having both parents born in a country other than Norway [18]. Living situation was dichotomized into “living with mother and father” or “all other living arrangements”. Age was measured in years.

### Statistical analysis

In addition to descriptive analyses, intra-class correlation coefficient analyses (ICC) were conducted to assess the test-retest reliability of the GFS. The reliability was classified as follows: “excellent” (≥ 0.81), “good” (0.61–0.80), “moderate” (0.41–0.60) and “poor” (≤ 0.40) [19]. Corrected Item-Total Correlations (CITCs) and Cronbach’s alpha were used to assess the internal consitency of the scale. CITCs > 0.30 were considered good, and CITCs < 0.20 were considered unreliable as it may indicate a lack of shared variance between some items included in a given scale [20]. Cronbach’s α > 0.70 was considered acceptable and α > 0.80 good [21].

A multiple logistic regression model was used to test for the association between family functioning and family dinners while adjusting for variables known to be associated with *family dinner* such as gender, age, parental education level, living situation and ethnicity. Data were analyzed using IBM® PASW® Statistics, version 20.0 (IBM Corp., Somers, New York, USA). The significance level was set to *p* < 0.05.

## Results

The sample characteristics are presented in Table 2. The adolescents were on average 14.3 years (*SD* = 0.6) and 52.3% were females. Most of the adolescents lived together with both parents (68.7%), while 31.3% had other living arrangements. In total, 66.2% of the adolescents’ parents had more than 13 years of education, and 90.9% were ethnic Norwegian. Most of the adolescents ate dinner together with their parent(s) 6–7 times per week (81.2%).

**Table 2:** Characteristics of the study sample

	Adolescents	
	Full sample N^a^ = 440	Test-retest N^a^ = 54
**Age** 13–15 year (mean (SD))	14.3 (0.6)	13.9 (0.3)
Gender (%)		
Boys	47.7	40.7
Girls	52.3	59.3
Dinner time together with parent(s) (%)		
0–5 times per week	18.8	17.3
6–7 times per week	81.2	82.7
Live together with (%)		
Mother and father	68.7	71.7
All other living arrangement	31.3	28.3
Ethnicity (%)		
Norwegian	90.9	88.7
Other ethnicity^b^	9.1	11.3
Parental educational level (%)		
< 12 years	33.8	9.3
13–16 years	39.3	37.0
> 16 years	26.9	53.7

^a^Adolescents; *n* = 417–440, test-retest sample; *n* = 53–54

^b^Other ethnicity: Both parents born in other country than Norway

Table 1 shows descriptive statistics and internal consistency of the GFS. The test-retest reliability was excellent (ICC = 0.83). The values of CITCs were good (> 0.40 for all items). The GFS had a high reliability, α = 0.85.

The multiple logistic regression model was statistically significant, χ2 [7], 26.634, *p* < 0.001, explaining 11% (Nagelkerke *R*^2^) of the variance in family dinner frequency (Table 3). Poorer family functioning was significantly associated with reduced frequency of family dinners after adjusting for the effects of gender, ethnicity, age, living situation and parental education (OR = 0.36, CI = 0.20–0.64).

**Table 3:** Associations between family functioning and frequency of family dinner in a sample of Norwegian adolescents

	Multivariable OR^b^ (95% CI)	*p-*value
Family functioning^a^	0.36 (0.20–0.64)	0.001
*Covariates*		
Gender	0.66 (0.39–1.13)	0.132
Ethnicity	0.36 (0.10–1.25)	0.107
Age	0.73 (0.45–1.17)	0.192
Living situation	0.60 (0.32–1.12)	0.108
Parental education^c^		0.024
Parental education (1) 13–16 y	1.80 (0.91–3.54)	0.090
Parental education (2) > 16 y	0.71 (0.36–1.39)	0.319
**Constant**	10,376.24	0.013
Nagelkerke R^2^	0.11	

^a^A higher score indicates poorer family functioning

^b^OR = Odds ratio

^c^< 12 years is the reference category. Parental education [1] = 13–16 years of parental education

Parental education (2) = more than 16 years of parental education

## Discussion

The GFS, assessing family functioning, had excellent test-retest and acceptable internal consistency in our sample of Norwegian 13–15-year old adolescents. Family functioning was significantly associated with family dinner frequency after adjusting for the effects of gender, ethnicity, age, living situation and parental education level. Importantly, a poorer family functioning was associated with a reduced odds ratio of having dinner together on a daily basis (i.e., 6–7 times per week).

Few studies have been identified assessing relationships between family functioning and family meals [6]. One study found an association between a healthier level of family functioning and more frequent family meals (both dinner and breakfast) in an American sample, which are in line with our findings [6]. These findings extend the result of a limited number of previous studies on family functioning and adolescent health [22, 23], as well as studies on family dinners outside America, showing that there are positive associations between family functioning and health behaviors such as having regular family dinners together.

The predictors in our model explained 11% of variance in family dinner. The modest amount of variance explained could mean that family functioning may be quite a distal factor, probably impacting on the relationships of more proximal family related variables (e.g., parenting style and more specific food parenting practices) [24]. Because the family and the home environment influence and shape adolescents’ dietary habits, parents play a major role in the development of healthful habits [3]. Thus, there is a need to explore family functioning, which can increase or decrease the likelihood of adolescents eating dinner together with their family.

### Strength and limitations

The present study is the first to use a Norwegian version of the GFS and test it among Norwegian adolescents to assess reliability and investigate associations between family functioning and family dinner frequency, thus expanding the research on such studies outside the US. The study has been reported according to the STROBE-nut guidelines [25] (see Additional file 1).

The existing literature on family functioning and family meal frequency is cross-sectional, as is this study, making it highly challenging to determine the direction of influence. Having regular family meals may promote a better family functioning, as well as the other way around. Additionally, the data was collected via self-report, which is prone to social desirability and recall bias. Having data from both parents and children could have strengthened the validity of the data. Other limitations are that the test-retest was conducted at one school in a high socioeconomic status area, and together with a low response rate, this will lower the generalizability of the findings. Finally, there is no information of the non-responders which may have caused bias in the study.

## Conclusion

The Norwegian version of the GFS used in this study showed high reliability in Norwegian adolescents aged 13–15 years. The association found between family functioning and family dinner frequency indicates that frequencies of family dinners could be one component to be targeted in interventions aiming to improve family togetherness and eating behaviors. Future research needs to investigate possible cause and effect between family functioning and frequency of family dinners by using longitudinal data and to relate it to the healthiness of meals. Furthermore, there is a need to explore more proximal family-related factors such as parenting style and more specific food parenting practices in relation to family functioning.

## Additional file

Additional file 1: STROBE-nut: An extension of the STROBE statement for nutritional epidemiology. This table provides a checklist, reporting adherence of the current study to the STROBE-nut guidelines. (DOCX 28 kb)
